# Complement dysregulation in human tauopathies

**DOI:** 10.1111/bpa.70017

**Published:** 2025-05-26

**Authors:** Jacqui Nimmo, Samuel Keat, Louis De Muynck, B. Paul Morgan

**Affiliations:** ^1^ UK Dementia Research Institute Cardiff Cardiff University Cardiff UK; ^2^ Janssen Research & Development Janssen Pharmaceutica NV, a Johnson & Johnson Company Beerse Belgium

**Keywords:** complement, frontotemporal dementia, inflammation, myelin, tau, tauopathy

## Abstract

Dysregulation of the complement system plays an important role in the pathogenesis of neurodegenerative diseases, including Alzheimer's disease (AD). In post‐mortem AD brains, complement is deposited in and around amyloid plaques, and peri‐plaque complement activation drives synapse loss in AD mouse models. Studies to date have focused on amyloid pathology; however, aggregated tau is also involved in neuronal loss in AD. Primary tauopathies are characterised by tau pathology in the absence of amyloid. The role of complement in human tauopathies remains largely unexplored. Here, we address this knowledge gap by assessing complement activation in human tauopathy brains using immunohistochemistry and well‐characterised detection tools. Post‐mortem pre‐frontal cortex was obtained from three tauopathy subtypes, Pick's disease (PiD), globular glial tauopathy (GGT) and corticobasal degeneration (CBD) (3–5 cases each). C1q and the complement activation markers iC3b and terminal complement complex (TCC) were assessed by immunohistochemistry and were elevated in all tauopathy cases compared to controls, with C1q and C3b/iC3b deposition particularly prominent on neurons, demonstrating complement activation on these cells. TCC deposits were present on and adjacent neurons in all tauopathy brains examined and were significantly increased compared to controls in CBD and GGT. Uniquely in GGT, abundant deposition of C3b/iC3b on myelin was also observed, implicating complement in GGT‐associated demyelination. To validate these findings, complement proteins (C1q, C3, factor B), regulators (factor I, clusterin) and activation products (Ba, C3b/iC3b, and TCC) were measured in brain homogenates by ELISA, revealing significant elevation in C3b/iC3b, Ba, and FI in CBD and GGT cases compared to controls. Together, our data demonstrate complement activation on and adjacent neurons in post‐mortem brains from all tauopathy subtypes.

## Graphical abstract

Complement protein and activation product levels were assessed in post‐mortem frontal cortex from three distinct subtypes of Tauopathy: Corticobasal degeneration, globular glial tauopathy, and Pick's disease. Quantification of complement proteins by immunohistochemistry and ELISA showed elevation of key complement proteins (C1q) regulators (FI) and activation products (C3b/iC3b, Ba, TCC), indicating dysregulation of the complement system in all tauopathy subtypes.

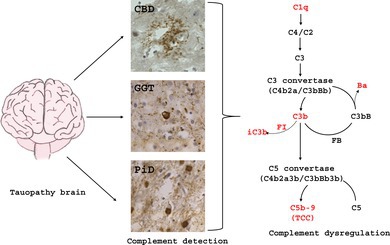

## 1 INTRODUCTION

Evidence implicating the complement system in neurodegenerative diseases (NDDs) has been accumulating over the last decade.^1^ To date, the focus has been on Alzheimer's disease (AD), and comparatively little is known about complement dysregulation in other NDDs, notably those that do not have amyloid pathology, including tauopathies. The tauopathies comprise a large group of NDDs characterised by the accumulation of hyperphosphorylated tau protein aggregates, predominantly in neurons and neurites but also in glia in some subtypes.^2^ Tauopathies are mainly sporadic in aetiology and are widely diverse in terms of clinical presentation, genetics and cellular pathology. These sporadic tauopathies include progressive supranuclear palsy (PSP), corticobasal degeneration (CBD), Pick's disease (PiD), argyrophilic grain disease (AGD), globular glial tauopathy (GGT), age‐related tau astrogliopathy (ARTAG), and primary age‐related tauopathy (PART).^2^ Aggregation of tau, the core feature of these tauopathies, is a frequent co‐morbidity in other NDDs, the most prevalent being AD, where it is considered an important player in neuronal death, contributing to dendritic spine loss and synaptic dysfunction.^3^, ^4^

Inflammation is a critical player in the progression of neurodegeneration in tauopathies; activation of microglia and astrocytes has been observed in post‐mortem studies,^5^ and is predictive of cognitive decline in longitudinal TSPO PET analyses of tauopathy cases.^6^, ^7^ The complement system is an important part of the immune system that, when dysregulated, causes uncontrolled inflammation and tissue damage in many contexts.^1^ In the brain, complement dysregulation can drive glial cell activation, tissue damage, synapse loss, and neurodegeneration. The roles of complement in dementia have mainly been studied in AD, focused on amyloid pathology as a trigger of complement activation. In mouse models of AD, complement activation drives synapse loss, the main correlate of cognitive decline.^8^ Similar findings have emerged from recent studies in P301S tauopathy mice, which show neuronal tau accumulation and involvement of classical and alternative complement pathways in synapse loss, with deficiency or inhibition of C1q or C3 being protective.^9^, ^10^, ^11^ In contrast, others reported that silencing of the complement C3 convertase regulator Crry in P301L tauopathy mice was protective, reducing neuroinflammation, the number of neurons with hyperphosphorylated tau, and subsequent neurodegeneration.^12^

Post‐mortem observations in AD brains have demonstrated the deposition of C1q, C3 fragments, C4, and terminal complement complex (TCC) in and around amyloid plaques.^13^, ^14^, ^15^, ^16^ Although some studies also report an association of complement with neurofibrillary tau tangles in AD brain,^13^, ^14^, ^17^ direct evidence for complement dysregulation in the brain in association with tau pathology is scarce. A few early studies explored complement deposition in post‐mortem brain from PiD and showed deposition of classical and terminal complement components on tau‐containing neurons.^18^, ^19^ These findings support observations of complement activation on neurons in P301S mice and suggest a role of complement in neurodegeneration in tauopathies in the absence of amyloid plaques. At the genetic level, a recent genome‐wide association study (GWAS) in a PSP cohort identified an association of PSP risk with the complement *C4A* locus^20^; of note, this same locus is a strong GWAS hit for schizophrenia, associated with increased expression of *C4A*.^21^

In order to clarify the extent of complement involvement in human cases of tauopathies across the various subtypes, we investigated complement proteins, regulators, and activation products in post‐mortem brains obtained from tauopathy patients with either CBD, GGT, or PiD. These subtypes differ in that PiD shows predominantly neuronal pathology, CBD has neuronal pathology with characteristic astrocytic tau plaques, while GGT is a relatively newly described disease subtype with a glial‐predominant pathology.^22^, ^23^ We show complement dysregulation in all tauopathy subtypes with different patterns in each. The data suggest that complement may be a significant contributor to pathology and a potential drug target in tauopathies.

## 2 METHODS

### 2.1 Tissue samples

Fresh frozen and formalin fixed tissue blocks from prefrontal cortex Brodmann area 8/9 (PFC, BA8/9) were obtained from well‐documented CBD, (*n* = 5), Pick's disease (PiD, *n* = 3), and GGT, (*n* = 5) donor brains (London NDD brain bank, Kings College London, UK; Table 1). Brain tissue samples from non‐demented donors matched for age and post‐mortem delay (*n* = 5) were used as controls. Formalin fixed tissue blocks were incubated in 30% sucrose at 4°C for 3 days for cryopreservation before embedding and freezing in OCT. Tissue blocks were then sectioned on a Cryostat (Cryostar NX‐50, ThermoFisher) at 10 μm thickness.

**TABLE 1:** Donor details.

ID	Age	Sex	PMI	Disease	Duration/years	Severity of pathology	Other pathological observations
BBN002.30838	73	M	35.5	CBD	4	Very extensive hyperphosphorylated tau and tau‐4R pathology.	Braak tangle stage II Thal phase 4 BNE stage II Mild CAA
BBN_10207	70	M	6.5	CBD	No data	Extensive tauopathy.	
BBN_4246	73	F	25	CBD	No data	Extensive tauopathy.	
BBN_15305	83	M	58	CBD	5	No data	
BBN_18397	84	F	25.5	CBD	8	Severe tau pathology.	Braak tangle stage III Braak LB stage 0 CERAD no plaques Thal phase 0 BNE Stage 3
BBN_15748	66	M	46	PiD	No data	Numerous Pick bodies and mild glial tau. Advanced atrophy of frontal lobes	No data
BBN_10248	80	F	90	PiD	13	Large number of Pick's cells. Severe atrophy of gyri and hippocampus.	Braak tangle stage 0 CERAD no plaques Braak LB stage 0 Thal phase 0
BBN_17591	66	M	54	PiD	6	Extensive tauopathy with abundant Pick bodies. Significant lobar atrophy.	Braak tangle stage IV CAA
BBN002.36900	71	M	52	GGT	No data	Extensive tau pathology. Severe cerebral atrophy	Braak tangle stage IV CERAD sparse neuritic plaques Thal phase 1 LB Pathology Negative
BBN002.38251	78	M		GGT	14	No data	No data
BBN002.32579	68	M	63	GGT	5	Extensive cortical and white matter tau pathology, Progressive cerebral atrophy predominantly in frontal lobes.	Braak tangle stage IV CERAD no plaques Thal phase 0
BBN002.26065	73	M	67	GGT	No data	Widespread tau pathology.	Mild CAA Braak tangle stage IV Braak LB stage 0 CERAD moderate Thal phase 1 BNE stage III‐IV
BBN002.28784	73	M	24	GGT	No data	Prominent tauopathy.	
BBN_9926	84	F	35	Control	N/A	N/A	Tau Braak stage II consistent with normal ageing
BBN002.28709	83	M	48	Control	N/A	N/A	Tau Braak stage II consistent with ageing
BBN_21005	76	F	22	Control	N/A	N/A	Tau Braak stage 2 consistent with ageing
BBN_16213	73	M	23	Control	N/A	N/A	Minimal age‐related tau. Normal brain.
BBN_10208	67	M	26	Control	N/A	N/A	Tau Braak stage 1, very mild ageing changes

Abbreviations: BNE, brain net Europe; CAA, cerebral amyloid angiopathy; CERAD, Consortium to Establish a Registry for Alzheimer's Disease; CBD, corticobasal degeneration; F, female; GGT, globular glial tauopathy; LB, lewy body; M, male; PiD, picks disease; PMI, post‐mortem interval.

### 2.2 Immunohistochemistry

Fixed tissue sections were dried and cleared in Xylene (10 min), rehydrated by sequential incubation in a dilution series of industrial methylated spirits (100%–50% in 4 steps; ThermoFisher, 10552904), then in phosphate‐buffered saline (PBS). Heat‐mediated antigen retrieval was performed in citrate buffer (10 mM citric acid, 2 mM EDTA, 0.05% Tween‐20, Ph 6) using a microwave (Russell Hobbs, 900 W) set at 50% power for 15 min, then left to cool for a further 15 min. The tissue was blocked in PBS/0.1% triton X (PBSt) containing 10% normal goat serum (NGS, Vector labs, S‐1000), 0.3% bovine serum albumin (BSA, Sigma‐Aldrich A2153‐500G) for 1 h at RT before incubating in primary antibody at 4°C overnight (Table 2). The tissue was then incubated in goat anti‐mouse or goat anti‐rabbit biotinylated secondary antibody for 1 h at RT followed by Avidin Biotin Complex (ABC, Vector Labs, PK‐6100) reagent for 1 h at RT. Chromogenic development was performed using the DAB kit (Vector labs, SK‐4105) using haematoxylin as a counterstain (Vector Labs, H‐3401). Tissue sections were dehydrated in industrial methylated spirits and Xylene before mounting in EcoMount (Biocare Medical, EM897L).

**TABLE 2:** Summary of antibodies used for immunohistochemistry.

Antibody	Target	Concentration	Supplier	Product code
C3‐30 (mAb)	iC3b	1:1000	In house	n/a
RabMab C3 (mAb)	C3	1:1000	Abcam	ab20999
C1q (pAb)	C1q	1:300	Dako	A0136
B7 (mAb)	C5b‐9	1:100	In house	n/a
PLPc	Myelin	1:200	Bio‐Rad	MCA839G
GFAP (pAb)	Astrocytes	1:1000	Dako	Z0334
Iba1 (pAb)	Microglia	1:1000	WAKO	019–19,741
AT8 (mAb)	Phospho‐Tau (Ser202, Thr205)	1:500	ThermoFisher	A11004
AH36‐FITC	Phospho‐Tau (Ser202, Thr205)	1:500	ThermoFisher	MA5‐45934

Abbreviations: GFAP, glial fibrillary acid protein; Iba1, ionized calcium binding adaptor molecule 1; mAb, monoclonal antibody; pAb, polyclonal antibody; PLP, proteolipid protein.

For double labeling of tau and complement components, tissue sections were incubated in BLOXALL endogenous blocking solution (Vector Labs, SP‐6000) for 10 min at RT. Sections were subjected to heat‐mediated antigen retrieval as described previously, blocked in 10% NGS, then incubated in mouse anti‐C3‐30 (anti‐C3b/iC3b; 1:1000) and rabbit anti‐tau (1:500) overnight at 4°C. Tissue sections were incubated in ImmPRESS alkaline phosphatase (AP) horse anti‐rabbit (RTU, Vector, Labs MP‐5401) and biotinylated goat anti‐mouse (1:500) secondary antibodies for 1 h at RT, followed by ABC for 1 h at RT. For C3‐30, DAB solution (Vector labs, SK‐4103‐400) was applied for 1.5 min, followed by AP blue substrate solution (Vector Labs, SK‐5300) for 4 min at RT. The sections were dehydrated in 50%–100% IMS, dried at 37°C, and dipped into xylene before mounting in EcoMount. Slides were imaged on the same day as mounting to prevent any leaching of the AP blue substrate out of the tissue.

For fluorescent staining, sections were incubated with primary antibodies as above, then with AlexaFluor‐conjugated secondary antibodies (1:500) with Hoechst stain (ThermoFisher; 1:1000) for 1 h at RT. Autofluorescence was quenched in 0.1% Sudan Black for 10 min at RT and washed in PBS/0.5% tween20 before mounting in FluorSave mounting media (Merck, 345,789). Slides were stored at 4°C in the dark until imaged.

### 2.3 Image acquisition and analysis

For DAB staining, whole sections were imaged using the ×20 objective on an Axioscan Z1 Slide scanner (Zeiss); scans were imported into QuPath for analysis of the percentage area of positive staining. For each case, 20 images each of 1650 square pixels (px^2^) were analysed within cortical layers III–IV, V–VI, and the subcortical white matter for AT8 immunostaining, or layer III–V only for the other markers. Because some tissue sections showed cryoartefacts, the area of positive immunolabeling was normalised to the tissue area, determined in QuPath, thus eliminating bias. Microglial dystrophy was assessed from Iba1 immunolabelled images using a modified semi‐quantitative method as previously described^24^ and illustrated in Figure S1. For double labeling of tau and C3b/iC3b using AP and DAB, whole sections were imaged using the x20 objective on an Axioscan Z1 Slide scanner (Zeiss). Scans were imported into QuPath, and the percentage of tau positive neurons colocalised with complement components was quantified using the manual counting tool. Up to 150 cells were counted in the cortex of each brain section, and the average number of cells was calculated from two consecutive brain slices.

Fluorescent images were taken on a Lightning confocal microscope (SP8, Leica Biosystems) at 40× magnification.

### 2.4 Preparation of brain homogenates

Fresh frozen tissue blocks (80–170 mg) from the grey matter of the PFC were homogenised in RIPA buffer (Pierce, 89900) supplemented with PhosSTOP protease inhibitor (Roche, 04906845001; 6% W/V ratio) using an automated Bead Bug microtube homogeniser (Sigma Aldrich). Homogenates were centrifuged at 1000*g*, 10 min at 4°C in a MicroCL 17R centrifuge (ThermoFisher) and the supernatant frozen at −80°C until use. Protein concentration in supernatants was determined using the BCA assay (ThermoFisher, 23235).

### 2.5 Enzyme linked immunosorbent assay (ELISA)

Complement proteins and activation products from classical, alternative, and terminal pathways were measured by sandwich ELISA as previously described and validated.^25^ Antibodies used in each ELISA are listed in Table 3. To determine a suitable dilution factor for measurement of each analyte in the brain homogenates, linearity experiments were performed for each ELISA to select a dilution where most samples fell within the linear portion of the log standard curve. NUNC Maxisorp 96 well ELISA plates were coated in capture antibody overnight at 4°C in bicarbonate buffer (pH 9.6). Plates were washed in PBS/0.05% tween‐20 (PBSt), blocked in 3% BSA for 1 h at 37°C, washed again, and brain homogenate or purified protein standards were added in duplicate in 0.3% (w/v) BSA in PBSt for 1 h at 37°C. All brain homogenates were measured immediately after thawing to avoid in vitro complement activation. Plates were washed, then HRP‐conjugated detection antibody (Donkey anti Ms/Rb 1:2000, Jackson Labs) or HRP conjugated streptavidin (R&D systems, 890803) in 0.3% BSA/PBSt was added for 1 h at 37°C. Plates were washed and the assay developed with OPD substrate (Sigma Aldrich, SIGMAFAST OPD, 1003427029). The reaction was stopped by adding 10% H_2_SO_4_, then absorbance was measured at 493 nm in a FLUOstar Omega Microplate Reader (BGM LABTECH), standard curves plotted, and the concentration of the analyte per ng of total protein was determined in GraphPad Prism. For Ba ELISA, samples were incubated overnight at 4°C, and all other incubations were conducted at RT; for TCC and Ba ELISA, detection antibody was biotin‐labeled and poly‐HRP streptavidin (ThermoFisher, 21140) used in the final step for additional signal amplification.

**TABLE 3:** Summary of antibody pairs used for ELISA.

ELISA	Antibody pair	Target	Concentration	Supplier
FB	Capture: FB28.4.2 (mAb)	FB/Ba	5 μg/mL	Gift from Santiago Rodriguez de Cordoba
	Detection: Gt anti FB (pAb)	FB/Ba/Bb	5 μg/mL	Comptech: A235
Ba	Capture: D22/3 (mAb)	Ba neoepitope	5 μg/mL	Hycult: HM2379
	Detection: P21/15‐biotinylated (mAb)	FB/Ba	1 μg/mL	Hycult: HM2254
C1q	Capture: 9H10 (mAb)	C1q	1 μg/mL	In house
	Detection: Rb anti C1q (pAb)	C1q	1 μg/mL	In house
FI	Capture: 7B5 (mAb)	FI	2 μg/mL	In house
	Detection: Rb anti FI (pAb)	FI	1 μg/mL	In house
TCC	Capture: aE11 (mAb)	C9 neoantigen	5 μg/mL	Hycult: HM2167B
	Detection: Biotinylated E2 (mAb)	C8	1:1000	In house
iC3b	Capture: Mab clone 9 (mAb)	iC3b	5 μg/mL	In house
	Detection: Biotinylated BH6 (mAb)	C3b/iC3b	1:500	Hycult: HM2168
C3 (intact)	Capture: 2898 (mAb)	C3/C3a	5 μg/mL	Hycult: HM2075
	Detection: HRP clone 3 (mAb)	C3d	1:1000	Hycult: HM2198

Abbreviations: FB, factor B; FI, factor I; Gt, goat; HRP, horse radish peroxidase; mAb, monoclonal antibody; pAb, polyclonal antibody; Rb, rabbit.

### 2.6 Aggregated tau measurement using meso scale discovery (MSD) immunoassay

A self‐sandwich Tau aggregate‐specific MSD assay using PT24 as coating and detection antibody was performed to quantify the levels of aggregated tau in brain homogenates. PT24 is an anti‐tau antibody binding to the N‐terminal epitope 23‐RKDQ‐26 on the tau protein and was generated during the same immunization campaign as the PT26 antibody described in Reference [26] The MSD plate (MSD, L15XA3) was coated with 30 μL per well of PT24 anti‐tau antibody at 0.5 μg/mL in PBS at 4°C overnight. The plate was washed three times in PBSt and blocked in 150 μL of 0.1% casein for 2 h at RT. After washing three times in PBSt, 25 μL of brain homogenates (5 μg per well in 0.1% casein) was added in duplicate; aggregated recombinant human tau protein was used as standard. The plate was incubated at 4°C overnight. After washing, 25 μL of sulfo‐conjugated PT24 detection antibody was added at a 1:1000 dilution in 0.1% casein for 2 h at RT. The plate was washed as before, and 150 μL of 2× Read Buffer T (MSD, R92TC‐1) was added to each well. The plate was read immediately on a MESO QuickPlex SQ 120.

### 2.7 Statistical analysis

Statistical analysis of IHC was performed on average values for each donor in GraphPad prism software. Shapiro–Wilk test for normality was first conducted on raw data values for each dataset. Where data presented a non‐normal distribution, a Mann–Whitney test was performed on independent groups of control and disease subtypes. For normally distributed data, one‐way ANOVA with Dunnett post‐hoc corrections was used to analyze the differences in complement levels between controls and disease types. Pearson correlation was used to identify relationships between complement markers and tau in both ELISA and IHC datasets and with microglia dystrophy in the IHC dataset. For correlations with tau, controls were excluded because all were completely negative for tau. Correlations were performed on grouped data from all tauopathy subtypes as there was no obvious difference in the distribution of datapoints or direction of correlation between the disease subtypes. Values that were below background levels or extreme values exceeding 1.5× the interquartile range were excluded from the analysis as indicated in the text.

## 3 RESULTS

### 3.1 Distribution of tau pathology and related gliosis in the prefrontal cortex (PFC)

**FIGURE 1:**
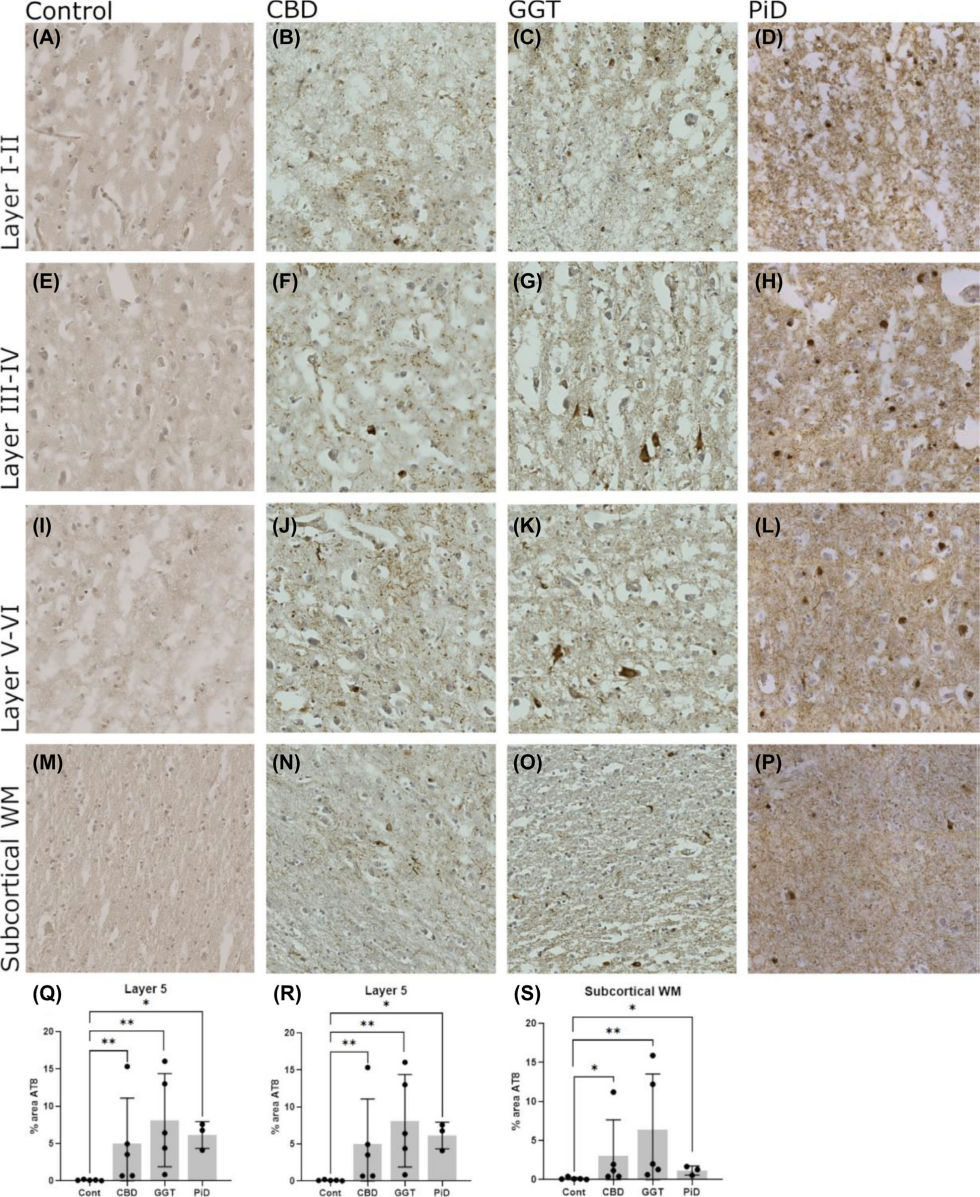
Tau pathology in the cortex of non‐demented control and tauopathy cases. Hyperphosphorylated tau (stained with AT8) was measured in cortical layers III–IV, V–VI, and subcortical white matter (WM) in control, CBD, GGT, and PiD. Controls were negative for AT8 immunoreactivity in all regions (A), (E), (I), (M). Image analysis demonstrated that tau signal (% area AT8 positive) varied between cases and layers, with tau most abundant in the pyramidal Layers (III–V) (B), (C), (F), (G), (J), (K), (N), (O), (Q), (S). Tau levels were consistently higher in GGT compared to other disease types in all layers, although not significant due to large variability between cases (Q), (R), (S). Control, *n* = 5; CBD, *n* = 5; GGT, *n* = 5; and PiD, *n* = 3. Error bars = mean ± SD. Scale bar = 100 μm.

Prefrontal cortex brain sections from CBD, GGT, and PiD cases were stained for hyperphosphorylated tau protein and markers for astrocytes (GFAP) and microglia (Iba1). The selected cases were at advanced disease stages (Table 1); all cases presented abundant tau pathology, as demonstrated in Figure 1. Controls were negative for hyperphosphorylated tau. Disease‐type characteristic tau inclusions were observed in all cases, including astrocytic plaques in CBD and Pick bodies in PiD. Two of the cases also presented sparse amyloid pathology (Table 1). The percentage area occupied by hyperphosphorylated tau (AT8‐positive) was measured across cortical layers III–IV, V–VI, and subcortical WM for each disease case, revealing large inter‐case variability. On average, the levels of tau pathology were similar between the tauopathy subtypes, with consistently higher levels in GGT across all cortical layers, albeit not significant because of the large intra‐group variability (Figure 1Q, R, S). In all subtypes, most of the tau pathology was observed in the main pyramidal cortical layers (III and V; Figure 1F, G, H, J, K, L), with consistently less tau in subcortical WM regions (Figure 1M, N, O, P). Subsequent analyses of complement and glial markers therefore focused on cortical layers III–V.

**FIGURE 2:**
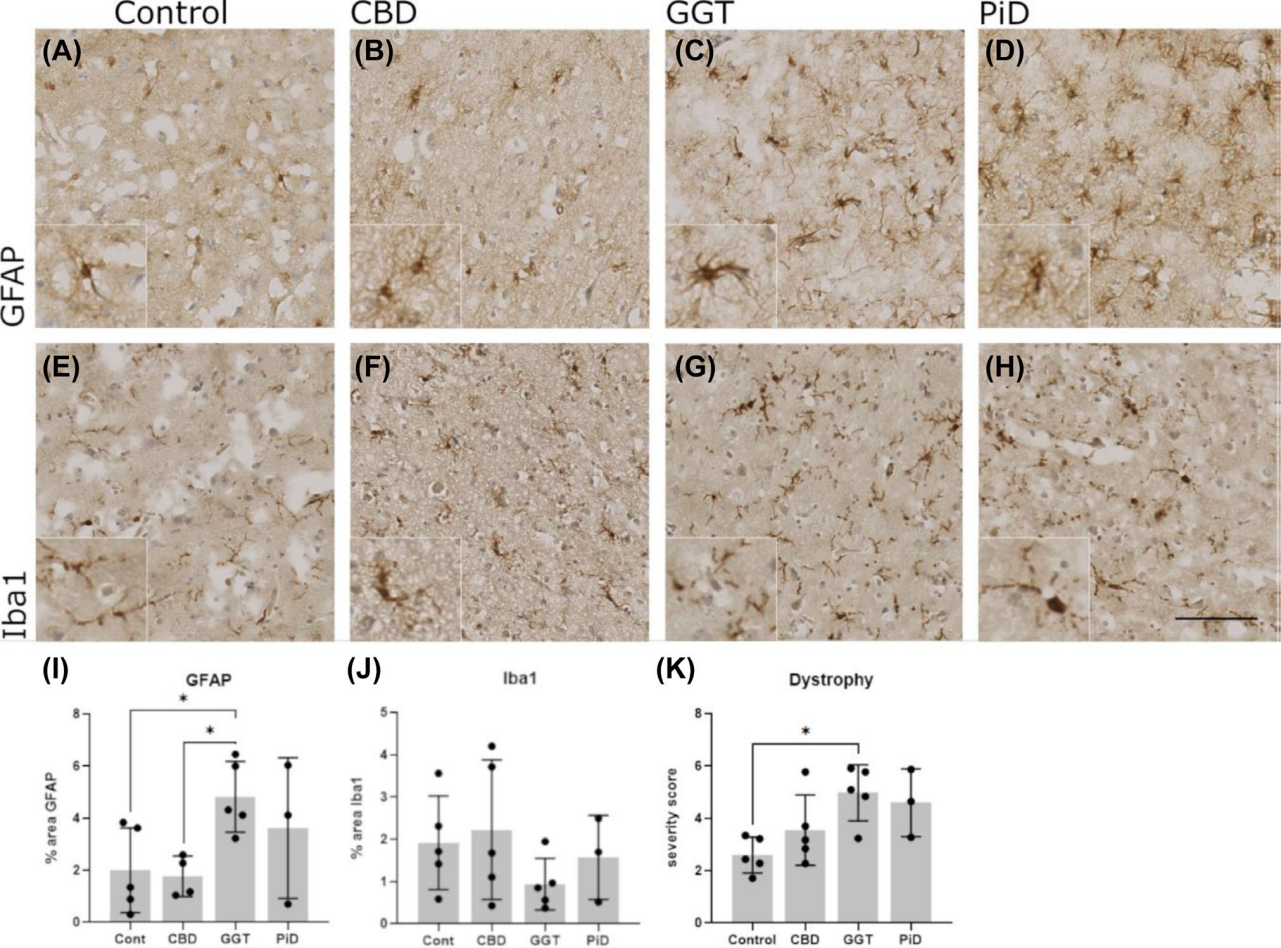
Glial cell activation in control and tauopathy. (A)–(D), Astrocytes were stained with anti‐GFAP in control, CBD, GGT, and PiD cases. (I) Quantification of % area GFAP immunoreactivity showed a significant increase in GGT compared to control cases (*p* = 0.0317). (E)–(H), Microglia were stained with Iba1 in control, CBD, GGT, and PiD. (J), quantification of % area Iba1 immunoreactivity did not show any significant differences in Iba1 levels. (K) Semi‐quantitative analysis of microglia dystrophy (E)–(H) showed a significant increase in severity in GGT cases compared to controls (*p* = 0.013). Control, *n* = 5; CBD, *n* = 5; GGT, *n* = 5; and PiD, *n* = 3. Error bars = mean ± SD. Scale bar = 100 μm.

Reactive astrocytes (using GFAP as marker; Figure 2A–D) were increased compared to controls in PiD and GGT cases, significant in the latter (Figure 2I; Mann–Whitney; mean: Control = 2.00, GGT = 4.822; *p* = 0.0317); in contrast, there was no difference in reactive astrocyte signal between CBD cases and controls (Figure 2I). Total microglia (using Iba‐1 as a marker) were not significantly different between tauopathy cases of any subtype or between subtypes and controls (Figure 2E–H, J). The lowest levels of Iba1 were observed in GGT cases, an unexpected finding given the reported gliosis in tauopathies.^6^, ^27^

To further investigate this unexpected finding, the extent of microglial dystrophy, previously reported as a feature of tau pathology,^24^ was analyzed in each disease subtype, scored using a modified semi‐quantitative method (Figure S1), as previously described.^24^ Mild dystrophy was observed in all controls (Figure 2E and K), consistent with the reported age‐dependent increase in microglial dystrophy^28^; moderate microglial dystrophy was observed in CBD and PiD (Figure 2F–H and K), whereas GGT cases all showed severe or very severe dystrophy scores, significantly higher than control cases (Figure 2G and K; one‐way ANOVA; Control = 2.60, GGT = 4.97; *p* = 0.013).

### 3.2 IHC reveals increased C1q, C3b/iC3b, and TCC deposition in tauopathy brains

**FIGURE 3:**
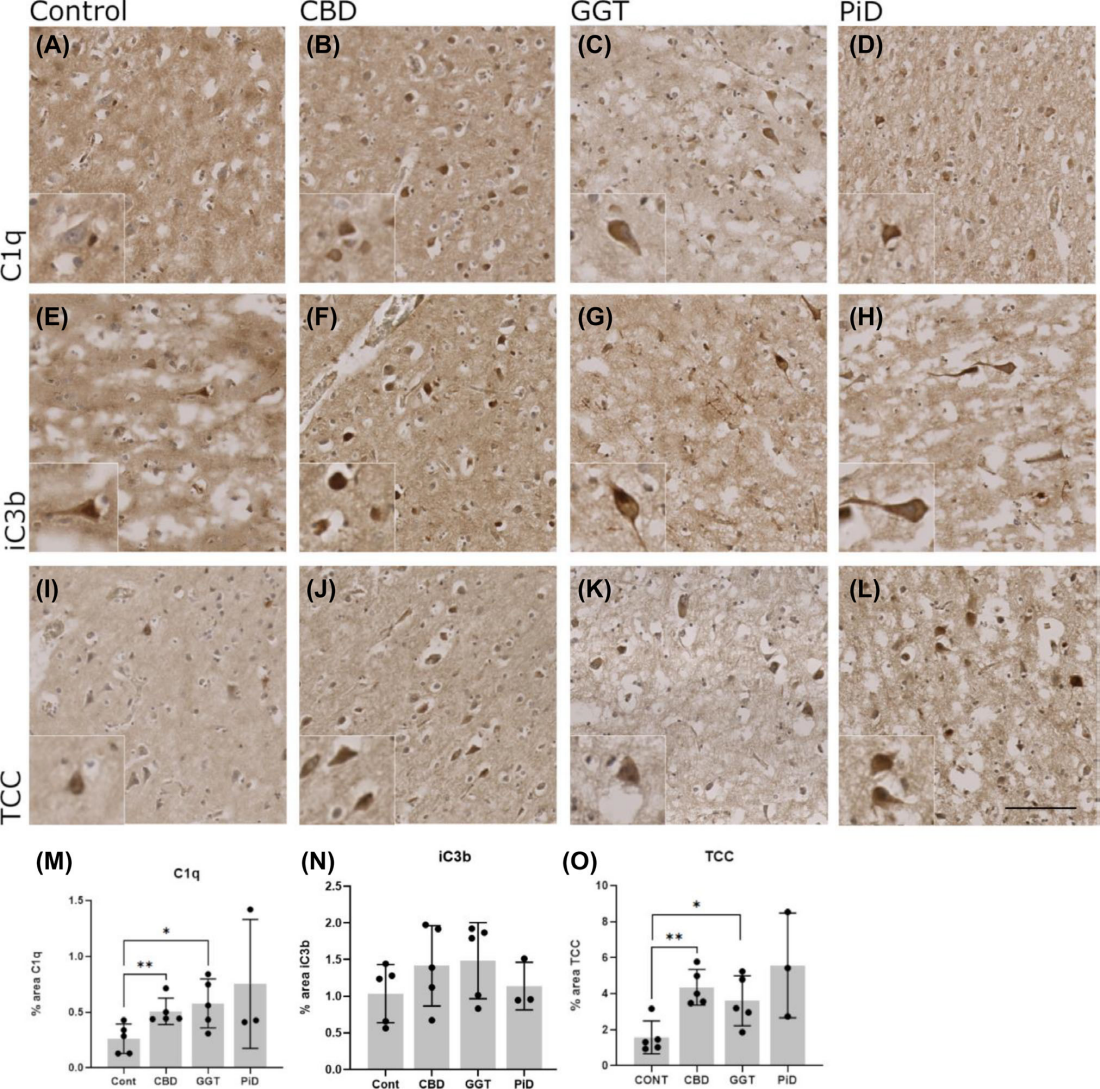
Immunohistochemical analysis of complement levels in non‐demented control and tauopathy cases. (A)–(D), C1q staining on neurons (inset) in control, CBD, GGT, and PiD cases. (M) Quantification of percentage area of C1q immunoreactivity showed a significant increase in CBD (*p* = 0.0079) and GGT (*p* = 0.0317) compared to controls. (E)–(H), C3b/iC3b staining on neurons (inset) in control, CBD, GGT, and PiD. (N) Quantification of percentage area C3b/iC3b immunoreactivity did not show any significant difference in C3b/iC3b levels in disease states compared to controls. (I)–(L), TCC staining on neurons (inset) in control, CBD, GGT, and PiD. O, quantification of percentage area TCC immunoreactivity showed significant elevation in CBD compared to controls (*p* = 0.0079) and GGT compared to controls (*p* = 0.032). Control, *n* = 5; CBD, *n* = 5; GGT, *n* = 5; and PiD, *n* = 3. Error bars = mean ± SD. Scale bar = 100 μm.

To determine the relative levels and distribution patterns of complement proteins and activation products in tauopathy cases compared to controls, the triggering protein C1q and the complement activation products C3b/iC3b and TCC were measured by IHC. Antibody specificity was validated for C1q and C3b/iC3b by pre‐absorption of the antibody with the respective purified protein. The percentage area coverage for each complement marker in each case was quantified in QuPath. The percentage area coverage of C1q staining was >2‐fold higher in all disease groups compared to controls, reaching significance for CBD (Mann–Whitney; mean Cont = 0.26%, CBD = 0.51%, *p* = 0.0079) and GGT cases (Mann–Whitney; mean Cont = 0.26%, GGT = 0.58%, *p* = 0.0317) but failed to reach significance in PiD cases (Figure 3A–D, M). C3b/iC3b percentage area coverage was also elevated in all tauopathies, approximately 1.5‐fold greater in CBD and GGT compared to controls, but not reaching significance (Figure 3E–H, N). TCC percentage area coverage was also markedly increased in all disease groups compared to controls, significantly increased in CBD (Mann–Whitney; mean Cont = 1.57%, CBD = 4.36%, *p* = 0.0079) and GGT (Mann–Whitney; mean Cont = 1.57%, GGT = 3.61%, *p* = 0.031) (Figure 3I–L, O).

To investigate the relationship between complement dysregulation and tau pathology or microglial dystrophy in the combined set of tauopathy cases, correlation analysis was performed for each complement analyte (C1q, C3b/iC3b, or TCC). No significant correlation with microglial dystrophy (Figure S2A–D) or hyperphosphorylated tau (Figure S2E–G) was identified with C1q, C3b/iC3b, or TCC.

### 3.3 Complement is deposited on tau‐positive neurons in all tauopathy subtypes and on myelin in GGT

**FIGURE 4:**
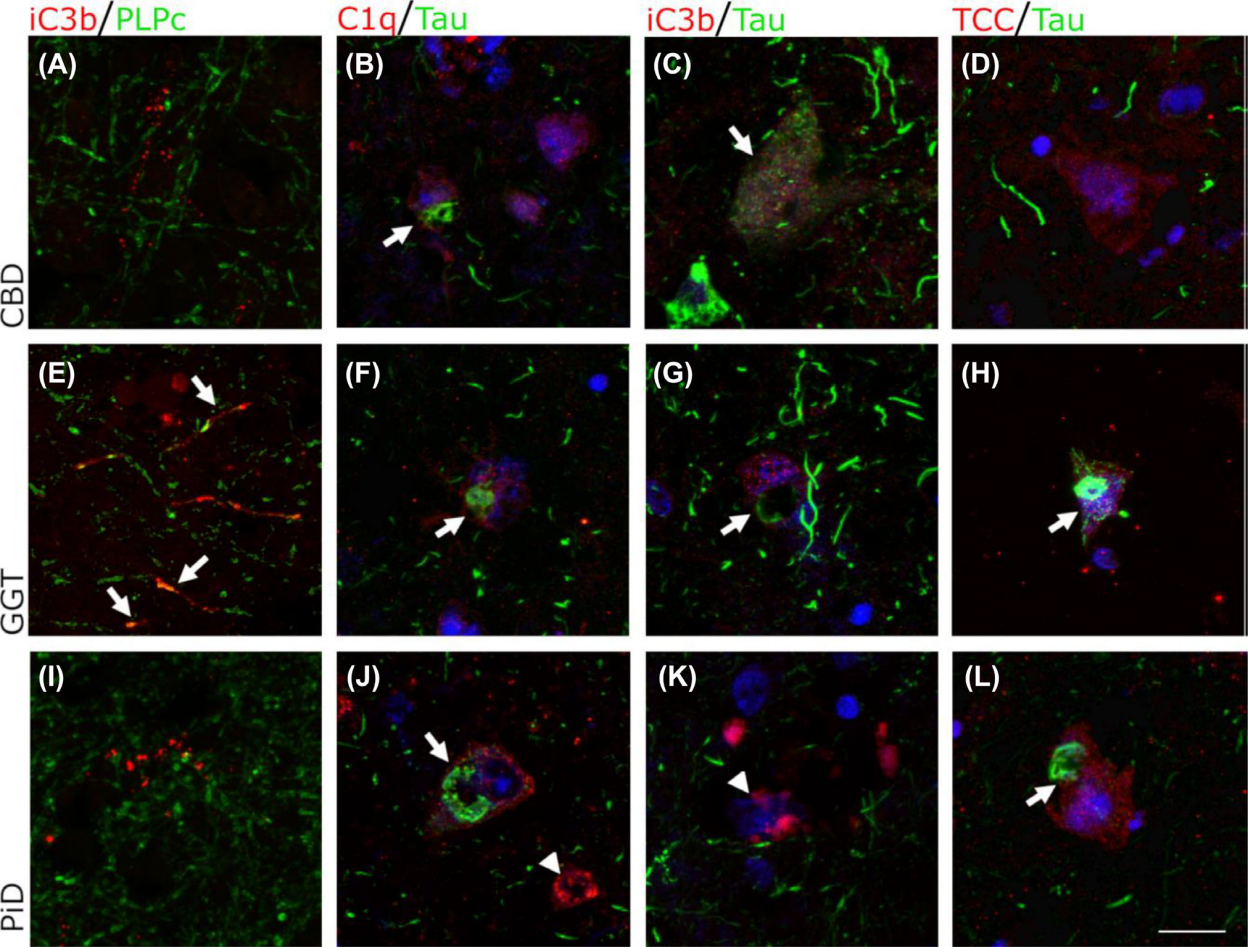
Co‐localisation of complement proteins with tau and myelin in CBD, GGT and PiD. (A), (E), (I): Double labeling of myelin marker PLPc (Green) with C3b/iC3b (Red). C3b/iC3b was observed co‐localised with PLPc in GGT cases only (E, arrows) but not CBD (A) or PiD (I). (B), (F), (J): Double labeling of C1q (red) with AT8 tau (green), Hoechst (Blue). C1q was observed on neurons both with tau (arrows) and without tau inclusions (arrowhead). (C), (G), (K): Double labelling of C3b/iC3b (red) with AT8 tau (green), Hoechst (Blue). C3b/iC3b was observed on some tau positive neurons in CBD (C, arrow) and on some tau positive cells in GGT (G, arrow). C3b/iC3b was also found on neurons in PiD that did not have tau inclusions (K, arrow). (D), (H), (L): Double labelling of TCC (red) with AT8 tau (green), Hoechst (Blue). TCC immunostaining was found in all tauopathy subtypes, including on neurons with tau positive inclusions (H, L arrows). Scale bar = 20 μm.

To more precisely identify the location of complement dysregulation in the tauopathy cases, immunofluorescence was performed on CBD, GGT, and PiD cases with double labeling for complement (the activation trigger C1q and activation products C3b/iC3b and TCC) and either tau (AT8) or myelin (PLPc). Representative images are shown in Figure 4. In all cases and all subtypes, complement activation markers (C1q, C3b/iC3b and TCC) were predominantly observed on and adjacent to tau‐positive neurons (Figure 4, arrows), compatible with activation on these cells. The proportion of tau‐positive neurons labeled with complement activation products was similar between groups; for C3b/iC3b deposition, this ranged between 33% in PiD to 45% in CBD (Figure S3). Complement activation markers were also found on some tau‐negative neurons (Figure 4, arrowheads). In GGT cases, C3b/iC3b was present on myelin, colocalized with the myelin marker PLPc (Figure 4E, arrowed); this was a consistent finding in four of the five GGT cases, but was absent in other tauopathy subtypes, implying complement involvement in the demyelination uniquely associated with GGT.

### 3.4 Tau and complement components are elevated in brain homogenates

**FIGURE 5:**
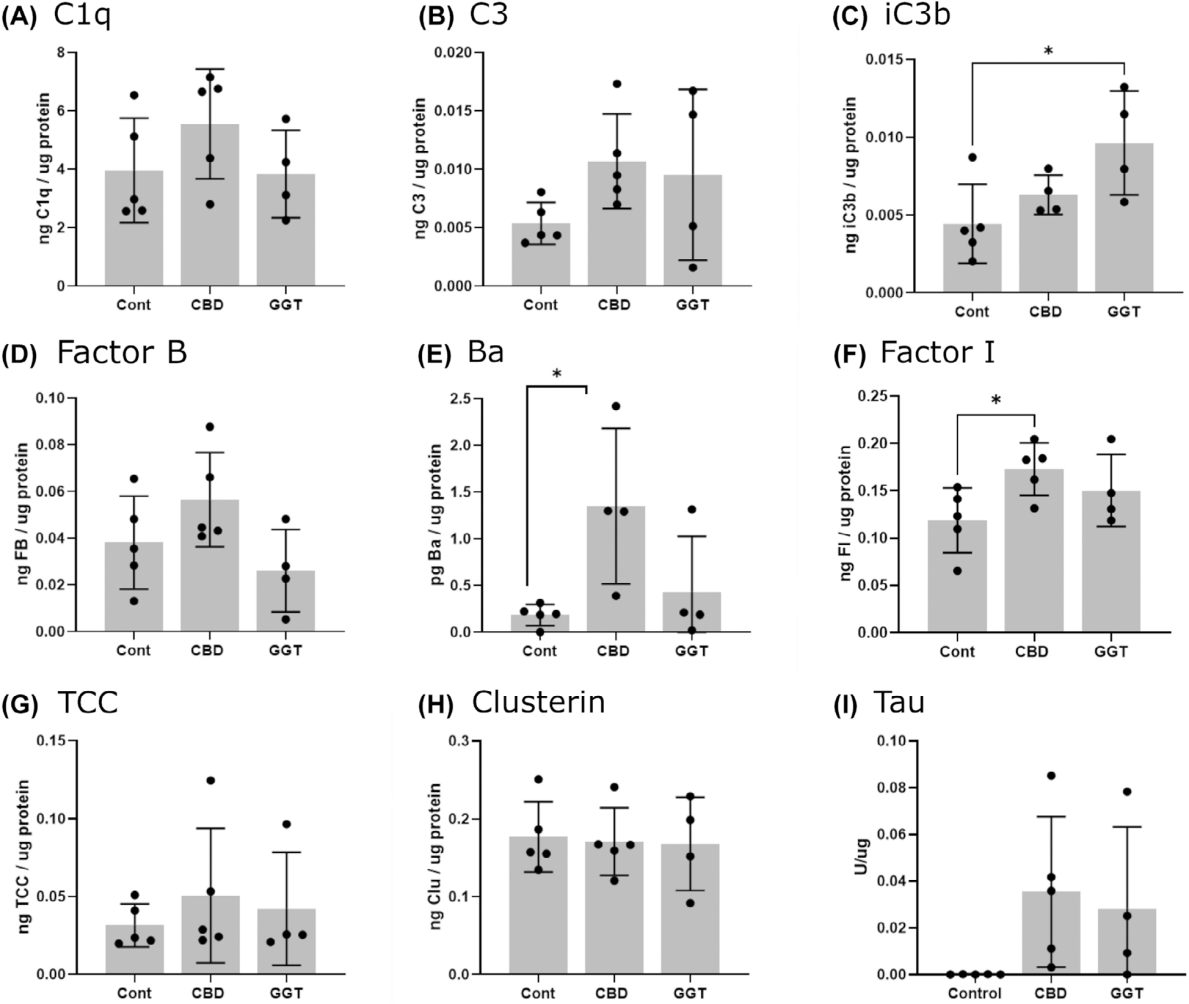
ELISA analysis for complement activation products in non‐demented control and tauopathy cases. Complement proteins and activation products were measured by ELISA in brain homogenates prepared from the grey matter of each brain sample. (A) C1q levels were comparable between control and tauopathy subtypes. (B) Total C3 levels were higher in CBD and GGT compared to control, although this did not reach significance. C, C3b/iC3b was elevated in all tauopathy subtypes and significantly increased in GGT compared to controls (one‐way ANOVA: *P* = 0.0024). (D) Levels of Factor B were comparable between cases, with slightly elevated levels in CBD, although not significant. (E) The Factor B activation fragment Ba was elevated in all tauopathy subtypes and significantly increased in CBD compared to controls (One‐way ANOVA: CBD, *p* = 0.0345). (F), Factor I levels were significantly increased in CBD compared to controls but not GGT (One‐way ANOVA: CBD, *p* = 0.049). (G) TCC was elevated in all tauopathies compared to controls, although not significant. (H) Clusterin levels were comparable across control, CBD, and GGT cases. (I) Aggregated tau was measured by MSD assay and confirmed that control cases were negative for aggregated tau, whereas all tauopathy cases possessed aggregated forms of tau. One value was excluded from Ba analysis because this was below background levels, and one extreme value was excluded from C3b/iC3b analysis as this exceeded 1.5× the interquartile range. Error bar = mean ± SD.

To further investigate complement dysregulation in tauopathy brain, quantitative sandwich ELISAs were developed and used to measure complement proteins (C1q, C3, FB), regulators (FI, clusterin) and activation products (C3b/iC3b, Ba, TCC) in grey matter brain homogenates, the same donor tissue used in IHC analysis (Figure 5). Both CBD and GGT cases showed a trend towards elevated levels of all complement analytes compared to controls, but these were not significant with the exception of FI and Ba in CBD cases (FI, one‐way ANOVA; mean Cont: 0.118, CBD: 0.173, *p* = 0.0457; Ba, one‐way ANOVA; mean Cont: 0.184, CBD: 1.350, *p* = 0.035) (Figure 5, E, F), and C3b/iC3b in GGT cases (one‐way ANOVA; mean Cont: 0.0044, GGT:0.0063, *p* = 0.0296). Only one frozen sample was available for PiD, so this was omitted from the statistical analysis and figures; however, this single PiD case showed a marked elevation in C1q, C3b/iC3b, FB, Ba, FI, TCC, and clusterin levels compared to controls.

Levels of aggregated tau in brain homogenates were assessed using an aggregate‐specific MSD assay (Figure 5I). Control cases were negative for aggregated tau, confirming the absence of tau pathology, whereas all tauopathy cases and all subtypes were positive for aggregated tau. There was considerable variability in levels of tau aggregates between donors in each subtype, consistent with what was observed in IHC (Figure 1). To explore the relationship between complement levels and tau pathology, correlation analysis was performed between each complement marker and aggregated tau (Figure S4). Significant positive correlations with aggregated tau levels were observed for FI (*r* = 0.82, *p* = 0.0065) and C1q (*r* = 0.69, *p* = 0.039) (Figure S4A, D).

## 4 DISCUSSION

Accumulating evidence from human genetics, biomarker and tissue analyses, and mouse disease models has firmly implicated complement dysregulation as a key player in neurodegeneration associated with AD and a direct cause of synapse loss, highlighting the therapeutic potential for targeting complement in AD‐associated neurodegeneration.^29^, ^30^ Although these studies have been focused on amyloid pathology as the driver of complement dysregulation, roles of tau are suggested by the demonstration of complement association with tau tangles in addition to amyloid plaques in AD cases.^13^, ^17^ Despite these clues implicating tau in complement dysregulation in AD, there remains a lack of knowledge of the role of complement in human tauopathies in which amyloid is absent. Early postmortem IHC studies in PiD demonstrated complement associated with tau pathology, while a study in tauopathy models in mice harbouring familial tau mutations replicated this complement association with tau.^9^, ^10^, ^11^, ^18^ Nevertheless, the relevance of complement dysregulation to tauopathies remains unclear, a particular deficit given the recognized contribution of chronic inflammation to their pathogenesis.^31^

Here we report a comprehensive investigation of complement dysregulation in tauopathies, including three human tauopathy subtypes with sporadic aetiology, CBD, PiD, and GGT. These disease subtypes all display distinct core features of tau accumulation but with unique aspects—astrocytic plaques in CBD, neuronal Pick bodies in PiD, and globular oligodendroglial inclusions in GGT.^2^ We focused our attention on three complement markers: C1q—the trigger of classical pathway activation and implicated in synapse loss; C3b/iC3b—activation products of C3 cleavage; and TCC—the activation product of the terminal pathway. These three mark targets of complement dysregulation in tissues. IHC analysis demonstrated deposition of C1q, C3b/iC3b, and TCC on and around tau‐positive neurons in CBD, GGT, and PiD, confirming that complement dysregulation is a core pathological feature of all three tauopathy subtypes.

Although staining for complement markers localised to tau‐positive neurons in the tauopathies (Figure 3A–D), the limitations of IHC in fixed tissue sections preclude a definitive localisation to the neuronal surface. However, because microglia are the sole source of C1q in the brain,^32^ its localisation on neurons strongly suggests localisation to the surface. The activation products C3b/iC3b and TCC would also be expected to decorate the surface of targets of complement dysregulation. Taken together, the localisation of all three markers on tau‐positive neurons strongly supports activation of complement on the membranes of these cells. The significantly elevated TCC staining on tau‐positive neurons indicates that complement was activated through to the formation of the terminal membrane attack complex, a potent driver of cell damage and death and a critical mediator of synapse loss.^8^

Not all tau‐positive neurons stained for complement activation products; quantification of C3b/iC3b staining showed ~40% of tau‐positive cells were labeled. We suggest that complement activation occurs on those tau‐positive neurons with exposed tau—either because their membranes are damaged, exposing tau, or because they are secreting tau in the process of seeding and propagation of tau oligomers. In support of this suggestion, others have reported that neurofibrillary tau tangles isolated from tauopathy brain directly bind C1q in vitro, activating the classical complement pathway in an antibody‐independent manner.^33^ In post‐mortem AD brains, extracellular tau tangles persist after degeneration of the neuron, known as ghost tangles. While these are less well characterized in tauopathies, it is reported that only a very small percentage of tangles (0.7%) represent ghost tangles even in advanced stages of AD (Braak V–VI).^34^ It is therefore unlikely that ghost tangles are solely responsible for the abundant complement staining we observe on tau‐positive neurons. In addition, some neurons that were negative for hyperphosphorylated tau were stained for complement. These neurons may represent those at early stages of neurodegeneration with the accumulation of tau oligomers invisible to IHC but visualized with more sensitive methods such as proximity ligation assays.^35^ To support the hypothesis that complement activation drives neurodegeneration in tauopathies, further quantification of complement labeling of intact tau‐positive and tau‐negative neurons and on extracellular tau at different disease stages is required.

The observed complement dysregulation was accompanied by pathological changes in microglia and astrocytes. Microglial dystrophy, morphologically associated with senescence and a sign of severe neurodegeneration, was a common feature of all tauopathies, as previously reported.^24^, ^28^, ^36^ Microglial dystrophy was particularly severe in GGT cases, which also showed significant astrogliosis. The high levels of microglial dystrophy explain the low levels of Iba1 staining in GGT cases and identify disease‐associated differences in microglia phenotypes in the different tauopathies. There was no significant correlation between microglial dystrophy and complement dysregulation, suggesting an indirect association in advanced stages of the disease.

A unique feature of GGT was the consistent finding of C3b/iC3b deposits on myelin, absent in CBD and PiD. This observation is consistent with the well‐described oligodendroglial phenotype of GGT cases with accumulation of globular tau inclusions in oligodendrocytes that likely impact oligodendroglial function and myelin integrity.^2^ Complement C3b/iC3b deposition on myelin and its resulting phagocytosis by microglia is a well‐described characteristic of multiple sclerosis^37^; our observation may suggest a mechanism of complement‐driven demyelination in GGT cases and other dementias where white matter loss is reported.^38^, ^39^, ^40^

We sought to confirm IHC findings using ELISA to measure complement proteins and activation products in brain homogenates from the same donors. Although levels of complement analytes were higher in tauopathy samples compared to controls, for example, a doubling of C3b/iC3b levels, most differences were not significant because of the small number of samples available. Nevertheless, the overall findings were supportive of the IHC study implicating complement dysregulation. Even with the small sample numbers, some analytes were significantly different for some subtypes, notably the activation product Ba and the core component FI, both elevated in CBD cases compared to controls, and C3b/iC3b elevated in GGT cases compared to controls; these findings suggest dysregulation in the alternative pathway. Surprisingly, TCC, the most significant difference between tauopathies and controls in IHC, was not significantly different in ELISA, although elevated compared to controls in most of the cases.

There are several limitations to our study. Most importantly, the small sample size, dictated by the difficulty in obtaining well‐validated brain tissue from these rare disorders; validation in independent cohorts is essential. As a further consequence, we only examined advanced stages of the disease, selecting donor brains displaying abundant hyperphosphorylated tau; longitudinal studies including various stages of the disease would provide a comprehensive understanding of the role of complement dysregulation in the pathogenesis of the disease. Despite these limitations, this study demonstrates that complement dysregulation occurs across the tauopathy subtypes and is not specific to amyloid pathology. The implication of complement dysregulation as a pathological driver in tauopathies raises the prospect of targeting complement activation as a therapeutic strategy, an approach that is already in development for AD.^30^

## AUTHOR CONTRIBUTIONS

JN conducted the experiments and prepared the manuscript, SK assisted with statistical analysis and manuscript preparation. BPM designed and managed the study, obtained essential resources, and contributed to manuscript preparation. LDM contributed to and reviewed the manuscript.

All authors read and approved the manuscript.

## CONFLICT OF INTEREST STATEMENT

The authors declare that they have no conflict of interest.

## ETHICS STATEMENT

This study received ethical approval from the London Neurodegenerative disease brain bank.

## Supporting information


**Figure S1:** Semi‐quantitative scoring of microglial dystrophy.
**Figure S2:** Correlation of complement and microglia dystrophy or tau.
**Figure S3:** Co‐localisation of C3b/iC3b and tau.
**Figure S4:** Correlation of complement and microglia aggregated tau.

## Data Availability

The data that support the findings of this study are available from the corresponding author upon reasonable request.
