# Supplementary material for: Complement dysregulation in human tauopathies
Source: Brain Pathol. 2025 May 26;35(6):e70017. doi: 10.1111/bpa.70017 (PMC12488255; doi:10.1111/bpa.70017)
Supplement: Supplementary file 1 — Figure S1: Semi‐quantitative scoring of microglial dystrophy. Figure S2: Correlation of complement and microglia dystrophy or tau. Figure S3: Co‐localisation of C3b/iC3b and tau. Figure S4: Correlation of complement and microglia aggregated tau. [file BPA-35-e70017-s001.docx]

**Figure S1: Semi-quantitative scoring of microglial dystrophy.**

Microglia dystrophy was scored based on the morphology of Iba1 immunoreactivity. 1, No dystrophy included microglia with intact branches reminiscent of resting phenotype. 2, mild dystrophy in which most microglia were normal with less than half having evidence of some dystrophy. 3, moderate dystrophy in which most microglia showed evidence of dystrophy but cell bodies were still present. 4, severe dystrophy where all the microglia were segmented in appearance, but some processes and cell bodies remained visible. 5, very severe dystrophy with few intact processes and cell bodies. 6, complete dystrophy in which there was no residual Iba1 present.

**Figure S2: Correlation of complement and microglia dystrophy or tau.**

Pearson correlation analysis to determine the relationship between complement levels and the extent of microglial dystrophy (A-C) or levels of hyperphosphorylated tau (E-G). The relationship between hyperphosphorylated tau and microglial dystrophy was also analysed (D).

**Figure S3: Co-localisation of C3b/iC3b and tau.**

A-C, Double labelling of hyperphosphorylated tau (Blue) and C3b/iC3b (C3-30, Brown) in CBD, GGT and PiD cases. D, The proportion of tau positive neurons with C3b/iC3b deposition (A-C, insets) were quantified within the cortex of CBD (45%), GGT (41%) and PiD (33%). There was no significant difference in proportion of cells between the disease subtypes. Scale bar = 100 µm, Error bars = mean +/- SD.

**Figure S4: Correlation of complement and microglia aggregated tau.**

Pearson correlation analysis to determine the relationship between complement levels in brain homogenate with aggregated tau. Significantly positive correlations were observed for A, C1q (r .68, P=0.039) and D, FI (r.82, P=0.0065).
